# Evaluating alternative temperature measurement sites in cats within a home environment: A comparison with rectal temperature

**DOI:** 10.1002/vms3.1423

**Published:** 2024-03-23

**Authors:** Dogukan Polat, Latif Emrah Yanmaz

**Affiliations:** ^1^ Department of Surgery Faculty of Veterinary Medicine Burdur Mehmet Akif Ersoy University Burdur Turkey

**Keywords:** axillary, auricular, eye, gingiva, metacarpus, temperature, thermal

## Abstract

**Objective:**

This study aimed to compare rectal temperature (RT) with temperatures measured in the pinna, cornea, medial canthus, gingiva, metacarpal pad and axillary region of cats in a home environment.

**Animals Studied:**

Five healthy mixed‐breed cats (two females and three males) owned by a veterinarian were used.

**Procedures:**

All temperature measurements were conducted by the owner by using an infrared camera in the same room and initiated with the pinna, followed by the cornea, medial canthus, gingiva and metacarpal pad. Subsequently, axillary temperature (AT) and RT were recorded by a digital thermometer, respectively. The time taken for a single AT and RT measurements was recorded.

**Results:**

The average measurement time for RT was 17.34 ± 0.89 s, with a range of 8–32 s, whereas AT measurements took an average of 46.72 ± 1.16 s, with a range of 29–69 s. AT emerged as a superior alternative measurement site compared to others, exhibiting the lowest bias and the highest proportion of readings within the limits of clinical agreement. The mean difference between RT and AT, with 95% limits of agreement for the differences, was −0.26 (−1.13 to 0.61).

**Conclusions:**

Anatomical regions were not all interchangeable with the rectum for assessing body temperature (BT), with AT recording the highest level of agreement with RT. When RT is not possible, AT could be considered as an alternative for monitoring BT in clinically healthy cats that live in a home environment.

## Graphical abstract

Graphical Abstract: In a home environment, temperatures from various anatomical sites in cats were compared to rectal temperature. Axillary temperature exhibited the closest agreement with rectal temperature, surpassing other sites in accuracy and reliability. With a shorter measurement time, axillary temperature emerged as a superior alternative, presenting itself as a viable option for non‐invasive body temperature monitoring in clinically healthy cats when rectal measurement is impractical.

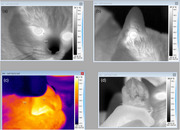

## 1 INTRODUCTION

Measuring body temperature (BT) is an important part of the routine physical examination and can provide important information to guide clinicians’ decisions (Sousa et al., 2011). Invasive contact devices such as oesophageal and pulmonary thermistors are the gold standard for assessing core BT, but these techniques can only be used in anesthetized animals (Greer et al., 2007). In most cats, BT is measured by placing contact thermometers against the rectal mucosa (Sousa et al., 2011). However, this technique can prove challenging as it may induce stress, particularly in irritable animals, and poses a risk of cross‐contamination if adequate hygiene measures are not observed (Kunkle et al., 2004). There is also a greater likelihood of rectal injury and discomfort in patients with pre‐existing anorectal and pelvic disease (Southward et al., 2006). Furthermore, although the rectal temperature (RT) shows relatively good agreement with core temperature, its accuracy and repeatability can be adversely affected by the depth of the probe, the presence of faeces, air and local blood flow (Kunkle et al., 2004).

Elevated BT can be attributed to various factors, including infection, inflammation, neoplastic growths, vigorous exercise, excessive heat exposure or heightened stress. Conversely, a reduced BT may be indicative of conditions, such as septic shock, inadequate perfusion, heart failure, exposure to cold temperatures or the effects of anaesthesia. Temperatures are also measured in healthy cats to ensure their suitability for elective procedures such as vaccination and spaying or neutering surgery (Levy et al., 2015). As changes in BT can occur due to environmental factors, stress or disease, temperature measurement in the home environment, in addition to the clinical setting, can provide valuable additional information about the cat's health status (Quimby et al., 2011).

Temperature evaluation from alternative anatomical sites, including the ear (Sousa et al., 2013; Nutt et al., 2016), axillary region (Smith et al., 2015), gingiva (Nutt et al., 2016), cornea (Giannetto et al., 2021) and metacarpal pad (Nutt et al., 2016), has been explored in cats. Auricular temperature exhibits limited concordance with RT (Kunkle et al., 2004; Garner, 2011). Likewise, prior investigations, as documented by Nutt et al. (2016), have established that gingival temperature (GT) and metacarpal pad temperature (MPT) are unsuitable for clinical use in feline patients. Conversely, corneal temperature displays a substantial agreement with RT, as highlighted in the work of Giannetto et al. (2021). In addition, axillary temperature (AT) may closely reflect RT in normal or underweight cats, compared to overweight cats, with good tolerance (Smith et al., 2015). To the author's knowledge, no study has yet investigated alternative temperature measurement sites to replace RT in cats within a home environment. Therefore, the aim of this study was to compare RT with temperatures measured from the pinna, cornea, medial canthus, gingiva, metacarpal pad and axillary region of cats in a home environment. Our hypothesis was that alternative anatomical sites, namely the pinna, cornea, medial canthus, gingiva, metacarpal pad and axillary region, could serve as reliable indicators of BT in cats within a home environment.

## 2 MATERIALS AND METHODS

### 2.1 Ethical approval

The study protocol received approval from the Local Board of Ethics Committee for Animal Experiments in Burdur Mehmet Akif Ersoy University (Decision No: 2023/1128).

### 2.2 Animal material

The study population comprised five mixed‐breed cats (two neutered females and three castrated males), all assessed as healthy based on examination by a veterinarian who was also the owner. The weight of the cats was 2–6 kg, and their ages were between 1 and 6 years old. The eldest cat had resided at home for 5.5 years, whereas the youngest had been there for 10 months.

### 2.3 Environmental conditions

All temperature measurements were conducted by the owner in the same room, within the owner's home. All procedures were performed out of direct sunlight in a consistent environment with an average temperature of 21°C (range 20–22°C) and a relative air humidity of 58.0% (range 51%–65%).

### 2.4 Measurements

**FIGURE 1:**
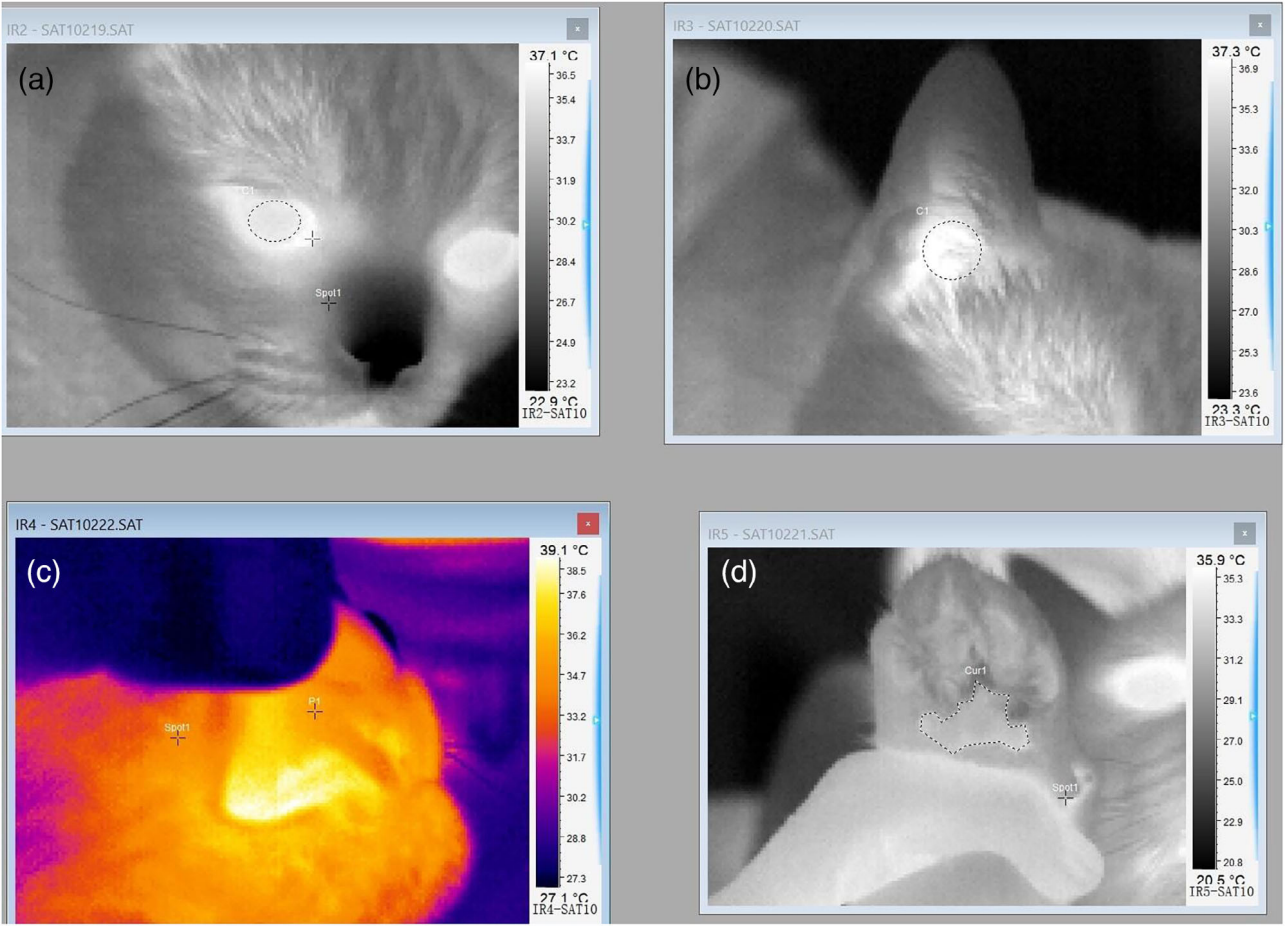
Images showing where measurements were taken in a study of cats comparing body temperature measured (a) using an thermographic camera at the corneal and medial canthus, at (b) the pinna, at (c) gingiva and at (d) the metacarpal pad.

Infrared temperature measurements were initiated with the pinna, followed by the cornea, medial canthus, gingiva and metacarpal pad. Subsequently, digital axillary and RTs were recorded, respectively. The reactions of the cats to the various measurement procedures were documented. A total of 308 temperature measurements were gathered, with 44 measurements recorded for each anatomical region (9 measurements for 4 cats and 8 measurements for 1 cat). These temperature measurements were obtained between 5 and 7 pm by the same individual, whereas cats were safely restrained by a co‐owner within the same household. The accuracy of the infrared thermal camera and digital rectal thermometer was verified by comparing them to a reference thermometer in a temperature‐controlled water bath before conducting the experiment. The infrared thermal camera (Trotec EC060V) was positioned at a distance of 30 cm from the region of temperature measurement. These areas included the right ear (for measuring the pinna temperature [PT]), right eye (for assessing the corneal temperature [CT] and medial canthus temperature [MCT]), the lateral right side of the mouth (after lifting the lips to measure the GT of the canine tooth gingiva) and the right palmar side of the extremity (for measuring the MPT) as illustrated in Figure 1a–d. The highest temperature point within each region was recorded as the temperature of interest.

All AT and RT were measured using a digital rectal thermometer (Kruuse Digital Thermometer; Jørgen Kruuse). The interval between the last infrared thermography measurement and the AT measurement was a maximum of 10 s. For axillary measurements, a digital thermometer was placed into the left axillary region from the caudal aspect with the extremity adducted and was kept in place until the audible endpoint beep sound was heard. The time taken for a single‐AT measurement was recorded. Following the axillary measurement, RT measurements were immediately obtained using the same thermometer. The thermometer was inserted into the rectum to a depth of approximately 2 cm and pressed against the rectal mucosa to ensure accurate readings and prevent intrafaecal temperature measurements. It remained in place until the audible endpoint beep was heard, and the time taken for a single‐RT measurement was also recorded. In the event of cats displaying signs of stress precluding RT measurement, the previously gathered data was excluded from the study.

### 2.5 Statistical analysis

The required sample size was determined using G*Power 3.1 with a significance level set at 5% and a power of 80% (Faul et al., 2009). This calculation indicated that 44 readings were required for each measurement, based on data from a previous study in dogs (Okur et al., 2022). Statistical analysis was performed using Medcalc version 20.011 (Medcalc Software). The differences between RT and the temperatures measured at other anatomic regions (AT, MCT, PT, CT, GT, MPT) were calculated. The normality of the differences (RT–AT; RT–GT; RT–CT; RT‐MCT; RT–MPT; RT–PT; and RT time–AT time) was confirmed using the Kolmogorov–Smirnov test and visualized using Q–Q plots. A paired sample *t* test was employed to compare the RT and AT measurement times. The agreement between RT and the tested alternative methods was evaluated using the Bland–Altman plot, in which the differences between the methods were plotted against their mean temperatures and the limits of agreement (mean ± 1.96× SD) (Bland & Altman, 1986). The results are presented as the mean difference and the 95% limits of agreement, along with appropriate measures of precision for each estimated parameter. For each comparison pair, a regression of the differences on the mean of the combined measurements was performed to assess the relationship between bias and the magnitude of the measurement (Bland & Altman, 1999). For each comparison method, the proportion of measurements falling within the limit of agreement was reported. Pearson's correlation coefficient *r* was computed to assess the relationship between RT and other anatomical regions. Data was presented as mean ± standard deviation.

## 3 RESULTS

**FIGURE 2:**
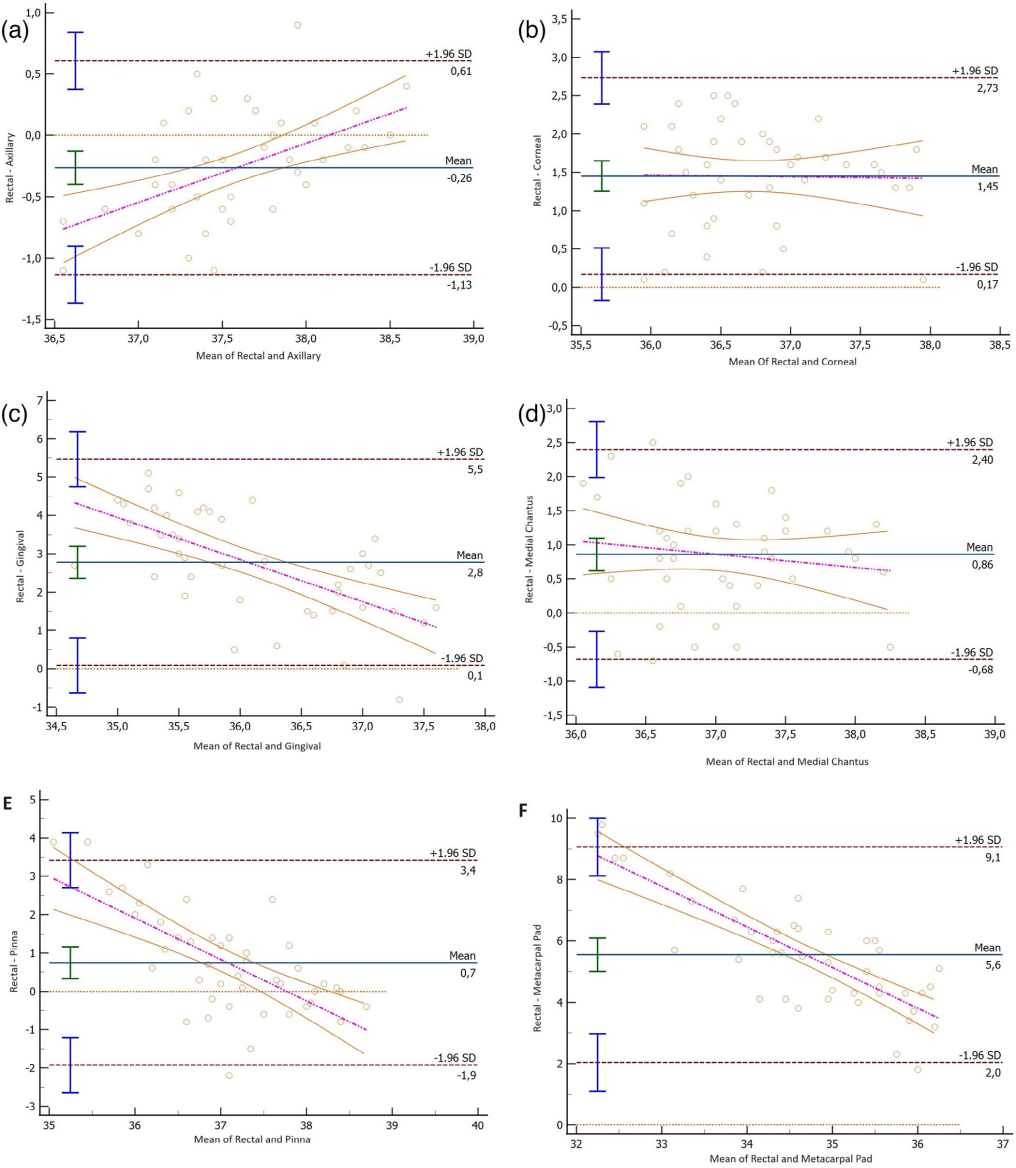
(a) Bland–Altman limits of agreement plots comparing axillary temperature; (b) corneal temperature; (c) gingival temperature; (d) medial canthus temperature; (e) pinna temperature; and (f) metacarpal pad temperature in healthy cats using rectal temperature as the reference. The solid black line represents the mean temperature difference, flanked by its 95% CI indicated by the dotted grey lines. Error bars on either side denote the precision of these estimates. Thick solid grey horizontal lines denote a pre‐defined clinical limit of agreement, set at ±0.5°C differences. The dotted horizontal line signifies zero difference. Additionally, the dash–dot sloping grey line illustrates the regression line's slope for the linear relationship between temperature difference and the mean of temperature, accompanied by its 95% CI in grey.

Ten temperature attempts were unsuccessful and were consequently excluded from the study due to intolerance of the cat to RT measurement. Temperature measurement at all other sites was well tolerated in all cats. In total, 308 temperature measurements were collected from various anatomical regions (including the rectum, axillary region, gingiva, cornea, medial canthus, metacarpal pad, and pinna) over the course of 5 consecutive days. The temperature measurement data is reported in Table 1. RT measurements all fell within physiologic limits, ranging between 36.7 and 38.9°C. AT measurements also remained within physiological bounds, ranging between 36.9 and 38.5°C. However, other measurement sites were not all within the physiological range. The average measurement time for RT was 17.34 ± 0.89 s, with a range of 8–32 s, whereas AT measurements took an average of 46.72 ± 1.16 s, with a range of 29–69 s. Among the various measurement sites evaluated, AT emerged as the superior alternative, displaying the lowest bias and the highest proportion of readings falling within the limits of clinical agreement. As RT serves as the gold standard, it was compared with temperature measurements from other anatomical sites to assess the mean deviation and 95% CI for each of these comparisons (Table 2) (Figure 2a–f). A strong correlation was observed between RT and AT (*r *= 0.7207), and a moderate correlation was observed between RT and CT (*r* = 0.4846). However, weak correlations were found between RT and other temperature regions (Table 2).

**TABLE 1:** Summary statistics for rectal, axillary, gingival, corneal, medial canthus, metacarpal pad and pinna temperature measured from cats.

Temperature (°C)					Mean ± SD time taken for the measurements (min‐max)
Measurement location	Mean (SD)	95%Cl	Median	Min, Max	
Rectal	37.45 (0.9)	37.26–37.64	37.4	36–38.8	17.34 ± 0.89 (8–32)
Axillary	37.71 (0.6)	37.59–37.84	37.8	36.9–38.5	46.72 ± 1.16 (29–69)
Gingival	34.67 (0.2)	34.26–35.08	34.45	32.7–37.7	ND
Corneal	36 (0.9)	35.8–36.19	35.95	34.9–37.9	ND
Medial canthus	36.59 (0.1)	36.37–36.81	36.65	35.1–38.5	ND
Metacarpal pad	31.88 (0.28)	31.3–32.46	32.1	27.4–35.1	ND
Pinna	36.7 (0.21)	36.27–37.13	36.95	33.1–38.9	ND

Abbreviation: ND, Not determined.

**TABLE 2:** Bland–Altman analysis comparing body temperature (°C) measured at differing regions with rectal temperature (RT) and results of regressing the temperature difference (*y*) on the mean temperature (*x*) to quantify changes in bias.

Difference (*y*) compared to RT						
Parameter estimate	RT–AT	RT–GT	RT–CT	RT–MCT	RT–MPT	RT–PT
Mean difference (95% Cl)	−0.26 (−0.39 to −0.12)	2.77 (2.36–3.19)	1.45 (1.25–1.65)	0.85 (0.62–1.09)	5.55 (5–6.09)	0.74 (0.33–1.16)
Limit of agreement (95% Cl)	−1.13–0.61	0.1–5.5	0.17–2.73	−0.68–2.40	2–9.1	−1.0–3.4
SE*_y_*_,_ *_x_*	0.11	0.2	0.18	0.21	0.14	0.18
Regression equation	*y* = −18.3984 + 0.4825*x*	*y* = 42.3718 + −1.0978*x*	*y* = 2.2603 + −0.022*x*	*y* = 8.0092 + −0.1931*x*	*y* = 51.5844 + −1.3274*x*	*y* = 40.7942 + −1.0800*x*
Slope coefficient (95% Cl)	0.48 (0.24–0.72)	−1.09 (−1.50 to −0.69)	−0.22 (−0.38 to 0.34)	−0.19 (−0.62 to 0.24)	−1.32 (−1.62 to −1.03)	−1.08 (−1.44 to −0.71)
*p*‐Value^a^	0.0003	<0.0001	0.9043	0.3742	<0.0001	<0.0001
*r* (*p*‐Value)	0.7207 (<0.0001)	0.2103 (0.1707)	0.4846 (0.0009)	0.3410 (0.0235)	0.3564 (0.019)	0.2943 (0.0525)

Abbreviations: AT, axillary temperature; CT, corneal temperature; GT, gingival temperature; MCT, medial canthus temperature; MPT, metacarpal pad temperature; PT, pinna temperature; *r*, Pearson's correlation coefficient.

^a^ Significance test for difference in slope from zero.

## 4 DISCUSSION

The primary focus of the current study was to compare GT, CT, MCT, MPT, PT and AT with RT in clinically healthy cats. We found that these anatomical regions were not all interchangeable with the rectum for assessing BT, with AT recording the highest level of agreement with RT. When RT is not possible, AT could be considered an alternative for monitoring BT in clinically healthy cats.

Although RT is considered to be the standard measurement to assess BT in conscious animals, it can be stressful to perform (Barton et al., 2022). Earlier research has noted that measuring RT in cats can be challenging, often causing stress, which may necessitate restraint during measurements (Kunkle et al., 2004). In our present study, we faced challenges in obtaining RT measurements in 10 instances, whereas cats displayed better tolerance for measurements in AT measurement. This aligns with findings from a prior study, suggesting that AT is better tolerated than RT recordings (Smith et al., 2015). Cats presented to veterinary clinics often exhibit heightened sensitivity to sound and stimulation. Additionally, ambient conditions, noise levels and social dynamics in such clinical settings may contribute to elevated stress levels in cats. Moreover, in the current study, measurements taken from owned cats by their veterinarian may lead to reduced stress levels, potentially resulting in more straightforward and expedited measurements.

To the best of the authors’ knowledge, no study has been undertaken to compare the duration of RT measurements with AT measurements. In this investigation, we observed a notable disparity in the average measurement times, with AT requiring significantly more time (46.72 ± 1.16 s) compared to RT measurements (17.34 ± 0.89 s). A prior study conducted in a veterinary clinic indicated a shorter total process time for AT measurement (36.59 ± 3.39 s) compared to RT measurement (39.03 ± 3.08 s). However, it is important to highlight that in the previous study, precise durations of measurements were not recorded. Instead, the entire process duration was calculated, with an additional 6 s included in the overall assessment (Beyer et al., 2023). Although we did not measure the time for each infrared measurement, it could be said that each of them took a similar amount of time. Additionally, it is notable that infrared measurements generally took less time than both AT and RT measurements. The extended duration of RT measurements compared to AT measurements in the current study may be attributed to the axillary area's increased exposure to external factors, such as air temperature. This exposure can potentially influence the time it takes for the thermometer to obtain an accurate reading. In contrast, the rectum offers a more stable and internally regulated environment, facilitating a faster and more accurate assessment of core BT.

In the current study, AT emerged as the superior alternative measurement site in comparison to GT, CT, MCT, MPT and PT. This preference is substantiated by AT's mean difference of −0.26 and its possession of the narrowest limits of agreement (−1.13 to −0.61). Our findings revealed good correlation and limits of agreement between RT and CT but indicated that CT measurements were approximately 1.45°C lower than RT measurements. Consistent with this finding, Giannetto et al. (2021) have previously recommended a downward adjustment of CT readings by approximately 1.19°C in comparison to RT measurements. Furthermore, our research aligns with the findings of Nutt et al. (2016), who identified GT, MPT and PT as unreliable options for clinical use in feline temperature assessment. Barton et al. (2022) have also stated that PT is not reliable for use in cats. Although MCT has been utilized in a prior study to assess stress levels in cats (Foster & Ijichi, 2017), its potential for evaluating BT in felines remains unexplored. A prior canine study suggested that MCT could serve as a means to assess RT (Yanmaz et al., 2015). Nevertheless, a separate study in 2022 explicitly warned against regarding MCT as a feasible substitute for RT measurements (Okur et al., 2022). Similarly, in the current study, MCT was not found to be interchangeable with RT, as evidenced by a mean difference of 0.85°C and a low limit of agreement ranging from −0.68 to 2.4°C.

Although it was strongly perceived that AT measurements induced the least stress during the process, it should be noted that this study did not attempt to quantitate the degree of stress using a scoring system. As it is likely to be clinically relevant, another limitation is that the study did not measure the total time taken for temperature measurement at each site. Comparison of temperature measurements of the same cats in clinical environment was not an aim of this study. However, it is worth mentioning that a prior study found no significant difference in the BT's of cats between home and clinic environments (Nibblett et al., 2015). Additionally, our study exclusively utilized normothermic cats. It is therefore crucial to acknowledge that the findings are not necessarily applicable to hyperthermic or hypothermic cats, and further studies would be required to specifically assess those patient populations.

## 5 CONCLUSION

In conclusion, the results confirm that not all anatomical regions can be used interchangeably with that of the rectum. Among the alternative locations tested, AT demonstrated the highest level of agreement with RT. Therefore, in situations where obtaining RT measurements is impractical, AT could be a viable alternative for monitoring BT in clinically healthy cats living in a home environment.

## AUTHOR CONTRIBUTIONS

*Conceptualization; methodology; validation; formal analysis; visualization; writing – original draft*: Dogukan Polat. *Conceptualization; methodology; writing – original draft; writing – review and editing; supervision*: Latif Emrah Yanmaz.

## CONFLICT OF INTEREST STATEMENT

The authors of this article have no conflicts of interest to declare.

## FUNDING INFORMATION

None.

### ETHICS STATEMENT

The authors confirm that the ethical policies of the journal, as noted on the journal's author guidelines page, have been adhered to and the appropriate ethical review committee approval has been received. The study protocol received approval from the Burdur Mehmet Akif Ersoy University Local Board of Ethics Committee for Animal Experiments (Decision No: 2023/1128).

### PEER REVIEW

The peer review history for this article is available at https://www.webofscience.com/api/gateway/wos/peer‐review/10.1002/vms3.1423

## Data Availability

The data that supports the findings of this study is available from the corresponding author upon reasonable request.
